# The burden of Chronic Pelvic Pain (CPP): Costs and quality of life of women and men with CPP treated in outpatient referral centers

**DOI:** 10.1371/journal.pone.0269828

**Published:** 2023-02-09

**Authors:** David Hutton, Aida Mustafa, Soha Patil, Saira Rathod, Gautam Shrikhande, Arnold Advincula, Jessica Drummond, Peter Gregersen, Jason Hall, Christine Metz, Alexandra Milspaw, Iris Kerin Orbuch, Peter Stahl, Amy Stein, Allyson Shrikhande

**Affiliations:** 1 Department of Public Health, University of Michigan, Ann Arbor, Michigan, United States of America; 2 Pelvic Rehabilitation Medicine Clinical Research Foundation, West Palm Beach, Florida, United States of America; 3 The Feinstein Institute for Medical Research, Manhasset, New York, United States of America; 4 Department of Obstetrics and Gynecology, Columbia University, New York, New York, United States of America; 5 Boston University Medical Center, Boston, Massachusetts, United States of America; 6 Dempsey Center for Digestive Disorders, Boston, Massachusetts, United States of America; 7 Advanced Gynecologic Laparoscopy Center, Los Angeles, California, United States of America; 8 Department of Urology, Columbia University, New York, New York, United States of America; 9 Beyond Basics Physical Therapy, New York, New York, United States of America; Dipartimento di Scienze Mediche e Chirugiche (DIMEC), Orsola Hospital, ITALY

## Abstract

**Introduction:**

Chronic Pelvic Pain (CPP) is a complex, multifaceted condition that affects both women and men. There is limited literature on the cost utilization the healthcare system and CPP patients incur. The purpose of this analysis is to characterize the overall healthcare utilization, cost burden, and quality-of-life restrictions experienced by CPP patients using data from an outpatient pelvic rehabilitation practice.

**Methods:**

Healthcare utilization data was gathered by systematically reviewing and analyzing data from new patient visit progress notes stored in the clinic’s electronic health records (EHR). We obtained in-network costs by using the FAIR Health Consumer online database. Overall costs were then calculated as the utilization times the per-unit costs from the FAIR database. Additionally, data on patients’ visual analogue scale (VAS), absenteeism, presenteeism emergency room visits, usage of common pain medications, use of diagnostics, and participation in common treatment modalities was gathered.

**Results:**

Data from 607 patients was used. The overall cost burden per patient for all surgeries combined was $15,750 for in-network services. The cost burden for diagnostics was $5,264.22 and treatments was $8,937 per patient for in-network treatments.

**Conclusion:**

Chronic Pelvic Pain was found to have a large cost burden of $29,951 for in-network services which includes treatments, diagnostics, and surgeries.

This analysis sets the stage for future investigations involving data on costs of medications that patients have tried prior to presenting to us and costs associated with work hours lost.

## Introduction

Chronic Pelvic Pain (CPP) is a complex, multifaceted condition caused by the complex interplay of gynecological, gastrointestinal, urological, musculoskeletal, neurological, and psychosocial conditions among others [1, 2]. American College of Obstetricians and Gynecologists describes CPP as noncyclic “pain symptoms perceived to originate from pelvic organs/structures typically lasting more than 6 months” or cyclical pain that has “significant cognitive, behavioral, sexual, and emotional consequences” [3]. Under the umbrella of CPP lies a multitude of predisposing factors that contribute to the development of the CPP pain complex (S1 Appendix). About 14% percent of women experience CPP during their life [4]. Urological chronic pelvic pain syndrome (UCPPS) affects 2%-16% of men worldwide [5]. The most common comorbidities in CPP and their prevalence include endometriosis (70%) [6], adenomyosis (46%) [7], fibroids (48%) [8], post-partum pelvic pain (44%) [9], IC (89%) [10], bladder pain syndrome (61%) [6], irritable bowel syndrome (39%) [11], anxiety (66%), and depression (63%) [12].

There is limited literature on the cost utilization the healthcare system and CPP patients incur. Direct costs associated with CPP, related organ system dysfunctions, and the indirect costs of productivity loss, absenteeism, and missed wages are significant. A majority of the cost burden data in CPP involves Endometriosis, a chronic systemic inflammatory condition typified by lesions of endometrial-like cells outside of the uterus which globally affects 1 in 10 women [13, 14]. Of the 170 million women suffering from Endometriosis [15], 71%-87% of patients suffer from CPP [16, 17]. In 2018, an evaluation of the cost burden of Endometriosis included managing pain symptoms via pharmacological agents and surgical interventions and wages lost from absenteeism. Their calculations demonstrated overall healthcare costs per patient per year as $16,573 [18]. A study found that Endometriosis generated a cost burden of $22 billion in the US in the year 2002 (78.6%: direct, 21.4%: indirect) [19]. In 2010, the economic burden was estimated to be $69.4 billion by analyzing 12 tertiary centers in 10 European Countries (32%: direct and 66%: indirect) [20]. This multi-center analysis did not take into consideration the additional costs on the healthcare system of physical therapy, behavioral therapy, Emergency Room visits, or outpatient treatment procedures. Prolonged symptoms and delayed treatment and diagnosis were associated with higher healthcare utilization [20]. Quality of life and physical functioning is affected by endometriosis. A study analyzing 5,879 women diagnosed with Endometriosis discovered a positive correlation between symptom severity and hours of employment productivity lost: women with mild severity reported a weekly loss of 1.9 hours compared to 15.8 hours lost for severe symptoms [21]. Women also suffer from worsening of quality of life with CPP, especially combined with endometriosis, due to difficulties related to pregnancy and obstetric outcomes. Endometriosis patients have significantly higher risks of preterm birth, miscarriage, placenta previa, small for gestational age, and cesarean delivery than women without the disease [22]. A 2021 systematic review of women with CPP calculated the direct yearly cost per woman to be between $16,970 to $20,898, this includes healthcare, prescription, and indirect costs (lost wages and reduced productivity) [23]. For men with CPP, a Northwestern University outpatient urology clinic calculated annual direct costs (via Medicare rates and non-Medicare reimbursements) and lost wages due to absenteeism. Using non-Medicare rates, direct costs were $6,534 per person. Average indirect costs through lost wages totaled $3,248 per person; the modest value reflects the exclusion of productivity loss while at work [24].

There is a strong psychosocial impact as CPP patients are affected by anxiety and depression at strikingly higher rates than the general population. A study analyzing 57 articles found women with CPP to be twice as likely to have depression (18.9% versus the control of 9.3%). Another study demonstrated anxiety to be more than four times as likely in CPP patients (29.7% versus 7%) [16] Depression and anxiety negatively affect the prognosis of CPP and determine the onset of symptoms as they can contribute to central sensitization and “priming of the nervous system” [25]. Mental health diagnoses have been shown to decrease work productivity through absenteeism and presenteeism [26]. The associated costs of depression and anxiety across 36 large countries is calculated to be $925 billion due to the estimated time of 50 million days of work lost due to the onset of depression and anxiety [27].

CPP patients often obtain insufficient relief of their symptoms, consult many doctors without obtaining a precise diagnosis/ appropriate management for many years, and can have the impression of being abandoned by the medical profession [28]. Currently, the treatment of CPP and its related comorbidities is a multimodal interdisciplinary comprehensive outpatient protocol involving pharmacological agents, physical therapy, behavioral health, lifestyle modifications of diet, exercise, and sleep as well as ultrasound guided peripheral nerve blocks, trigger point injections and/or surgical interventions if conservative management fails to resolve symptoms [29, 30]

CPP represents a significant individual and societal burden and although researchers discuss the substantial psychosocial and economic impact, a precise figure is difficult to determine due to the lack of multidisciplinary studies and limited understanding/consensus regarding CPP among researchers and health providers [31]. The purpose of this analysis is to characterize the overall healthcare utilization, cost burden, and quality-of-life restrictions experienced by CPP patients using data from an outpatient pelvic rehabilitation practice.

## Materials and methods

This analysis evaluates costs CPP patients incur before they present to an outpatient pelvic rehabilitation practice treating CPP. The practice has 13 locations in large metro areas across the United States. To evaluate costs, we gathered healthcare utilization data from patient medical records and histories and combined the utilization data with unit costs. This was a retrospective chart review of medical records. All data was fully anonymized with medical record numbers prior to our research team having access to the data. The systemic utilization of specific medical and surgical treatments within the patient pool was recorded by our physicians while collecting patients’ medical history at new patient visits. The medical and surgical treatments identified are procedures patients have undergone prior to presenting to the pelvic rehabilitation clinic. So, healthcare utilization data was gathered by systematically reviewing and analyzing data from new patient visit progress notes stored in the clinic’s electronic health records (EHR).

In addition, we reviewed the progress notes to obtain information regarding the indirect costs of CPP and related comorbidities, including those associated with reduced quality of life measures, reduced work and school productivity, and increased disutility of care. In this paper, disutility of care refers to usage of common pain medications prescribed for pain management, including NSAIDs and Opioids, and ER usage. This information is not a direct factor in analyzing the cost burden associated with CPP in this paper, but rather reviewed to facilitate supplementary discussion around common indirect costs incurred by CPP patients.

### Patient pool selection

Patients were selected as new patients first seen from between April 5^th^ 2021 to June 18^th^ 2021. Patients were deemed eligible if they reported a duration of pain or discomfort for 6 months or longer. Patients medical history data was exhaustive, all previous surgeries, diagnostics, and treatments tried were recorded and used in this analysis. Previous medications and ER visits were limited to the past 12 months.

To quantify quality of life challenges our patient pool suffered from prior to presenting as New Patients, we analyzed their visual analogue scale (VAS), absenteeism, presenteeism emergency room visits, usage of common pain medications, use of diagnostics, and participation in common treatment modalities. The VAS is a self-reported measure which asks, “How would you rate your average pain or discomfort on a scale of 0–10?”. The patient reports the average pain they experienced in the past week before presenting as a new patient. Absenteeism and presenteeism are determined by the following questions: “How many days of work did you miss in the past 3 months due to your pain or discomfort?” and “On average, how many hours a week is your work productivity affected due to your pain or discomfort?”, respectively. Although associating costs with absenteeism and presenteeism is not conducted in this analysis, we gathered this data to calculate the hours our patients are losing. Data on ER visits is contained to the past 12 months and are only CPP- specific ER visits. Data on medications are also limited to the past 12 months while previous surgeries, diagnostics, and treatments data was lifetime.

### Systemic utilization criteria selection

After overall procedures were identified, seventeen types of surgeries were selected for analysis within this patient pool. Of these surgeries, nine were female specific, two were male specific, and six were gender neutral. These surgeries were selected given they are reported as common surgeries undergone by CPP patients [32, 33]. Moreover, ten types of diagnostics and one distinct previous treatment were selected for analyzation. These diagnostics vary across specialties, including Urology, Gynecology, Colorectal, amongst others. Pelvic floor physical therapy was selected as the treatment to be analyzed as it was most utilized by our patient pool.

### Cost association of systemic utilization

We obtained in-network costs by using the FAIR Health Consumer online database. FAIR Health is an independent, national nonprofit organization which provides information about healthcare costs. Their database includes more than 34 billion private health care claims and 36 billion Medicare claims for 10,000 services in all areas of the United States. FAIR Health uses this data to estimate what providers charge and what insurers pay [34].

Because FAIR Health requires input of geographic location to determine cost estimate per CPT code, we used 10001 (New York, New York) given our patient pool comes from large metropolitan areas, with the majority coming from New York, New York. The CPT codes along with the costs associated can be found in Table 1. Overall costs were then calculated as the utilization times the per-unit costs from the FAIR database.

**Table 1:** Procedures and their associated CPT codes.

Procedures	CPT Code
**Treatments**	
Pelvic floor physical therapy	90901
**Diagnostics**	
Ultrasound	76830
MRI	72197
Exploratory Surgery	49320
Urodynamics	51741
Sigmoidoscopy	45330
Semen Culture	89320
Anal Ultrasound	76872
Colonoscopy	45378
Endoscopy	43235
Cystoscopy	52000
**Surgeries**	
Hysterectomy	58150
Endometriosis excision	58662
Endometriosis ablation	58563
Oophorectomy	58661
Ovarian cystectomy	58925
Myomectomy	58545
Pelvic floor repair	57425
Bladder sling	57288
Uterine artery embolization	37243
Varicocelectomy	55550
Testicular torsion surgery	54600
Inguinal hernia repair	49650
Hemorrhoid surgery	46260
Colorectal surgery	44160
Other hernia repair	49652
Lumbar spine surgery	63030
Hip FAI / Labral tear repair	29916

## Results

### Patient population

**Fig 1:**
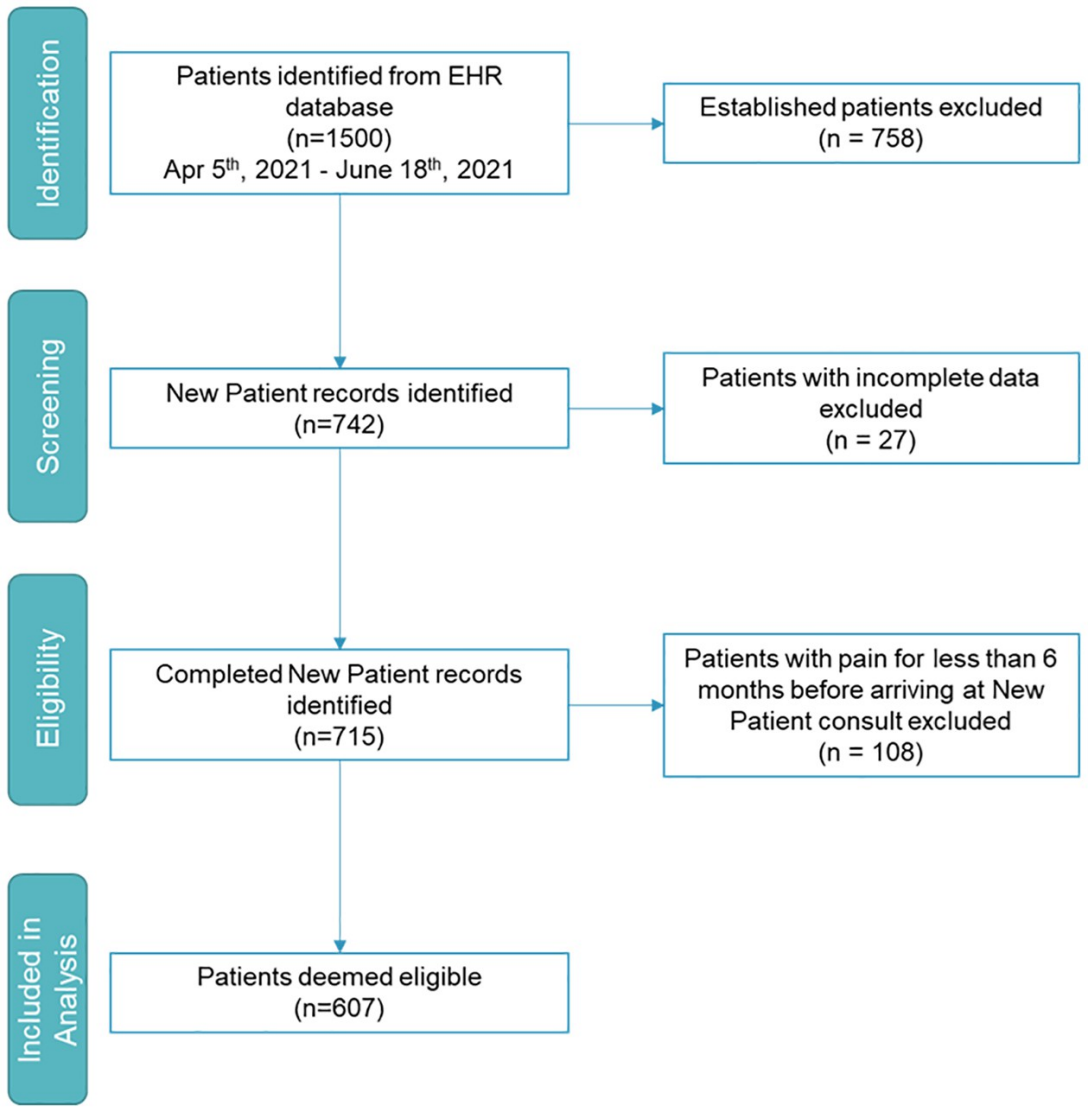
Study design.

We initially selected 742 CPP patients from our records who were seen as new patients in 2021. All patients were assessed for eligibility whereas 27 were excluded because they did not report the duration of pain or discomfort; and 108 were excluded for reporting pain with a duration of less than 6 months. This concluded in 607 patients analyzed for this study. Patient demographics, including sex at birth, and age, did not influence eligibility of inclusion (Fig 1).

### Patient demographics and clinical characteristics

**Fig 2:**
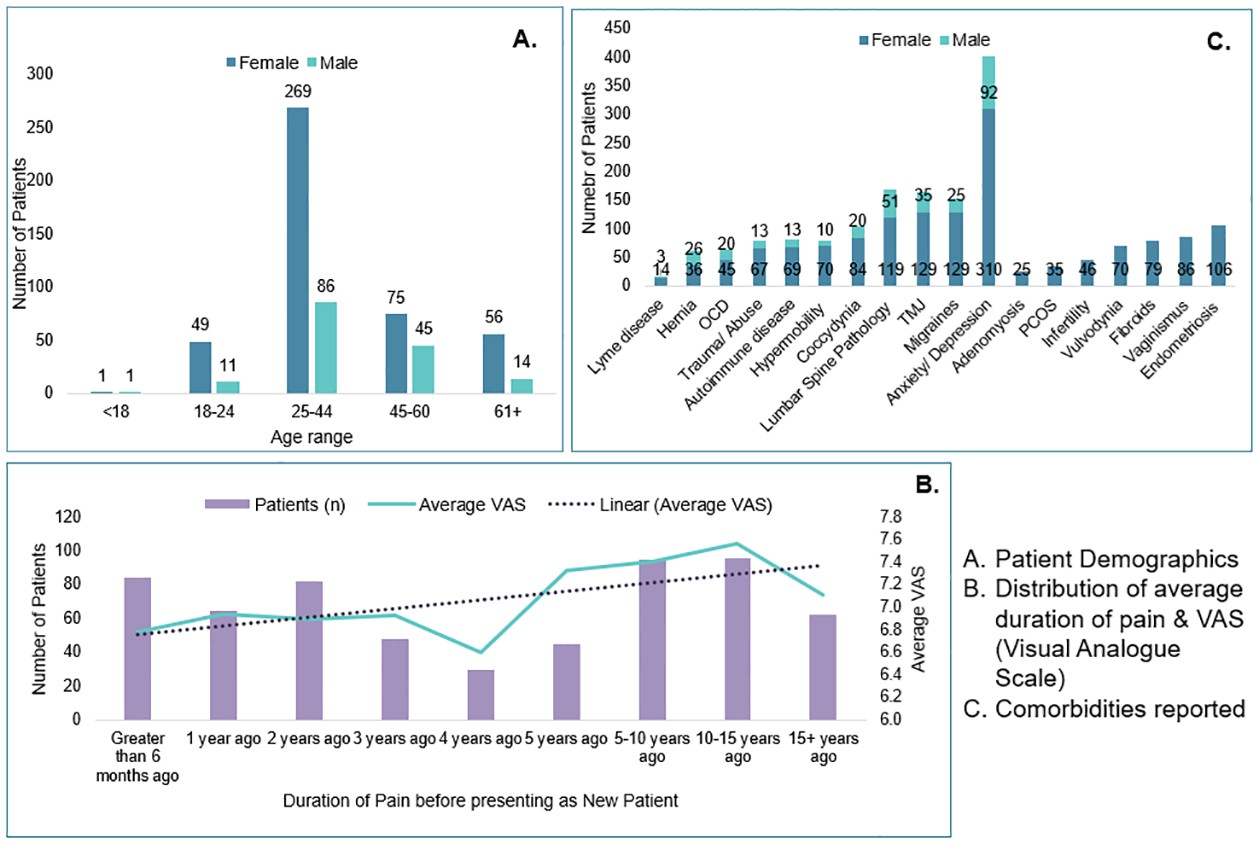
Patient demographics and clinical characteristics.

The demographic breakdown of the patient pool resulted in 74% female (450), and 26% males (157), with the age range between 14–88 years old. Less than 1% of the patient pool was younger than 18 years old; 68% were between 18–44 years old; 20% were between 45–60 years old, while 12% were above 61 years old (Fig 2a). The average Visual Analogue Score (VAS) score amongst all patients at the initial New Patient visit was 7.1 on a scale from 0–10. Every patient reported pain or discomfort with CPP having started greater than 6 months to being seen, while 50% of patients reported pain or discomfort having started 5 or more years ago. Amongst these longer-term patients, 10% of patients reported pain or discomfort starting more than 15 years ago. A breakdown of the average duration of pain and associated VAS scores can be found in Fig 2b. Furthermore, since this patient population is prone to comorbidities and overlapping pain syndromes, we analyzed the comorbidities in our patient population and found 93% of patients suffer from at least one comorbidity and the highest frequency of comorbidities are: Anxiety/ Depression (71% among patients with at least one comorbidity) and Endometriosis (24% among women). A complete list of comorbidities reported by patients when they presented to us as New Patients is shown in Fig 2c.

### Utilization of procedures

**Fig 3:**
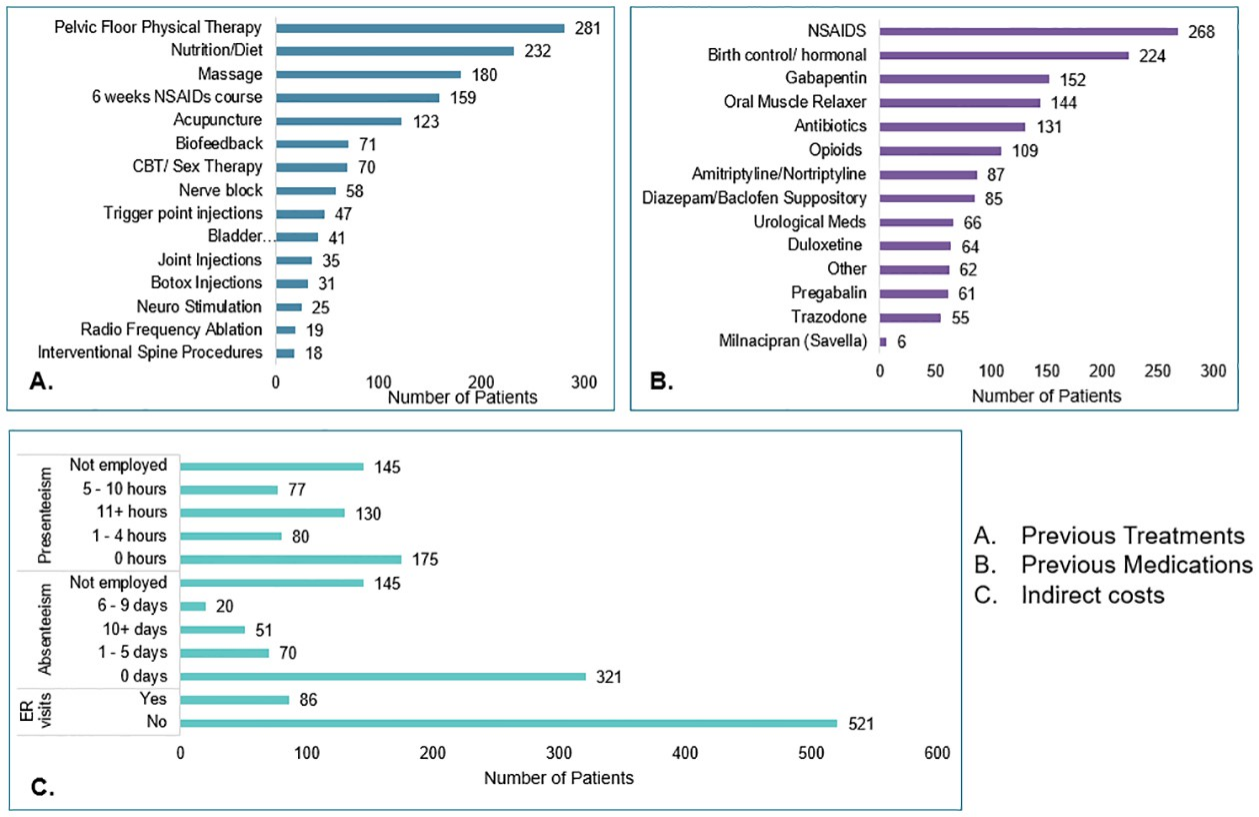
Procedures, medications, and productivity.

Non-pharmacological treatments are usually recommended first due to their ease in implementation and potential for long term maintenance compared to pharmacological and surgical options. Pelvic floor physical therapy for internal and external myofascial release, visceral mobilization, nerve gliding, as well as balance, movement, and neuromuscular re-education is recommended as first line therapy [35]. This is supported by our data which shows physical therapy was the most common treatment our patients had prior to consulting with us. 46% of patients attended at least 1 physical therapy session (Table 1 and Fig 3a). Out of this patient pool, each patient averaged 24 completed physical therapy sessions prior to being seen. On the higher end, one patient reported having completed 500 physical therapy sessions while still experiencing pain and discomfort with CPP. Details on the number of patients who tried specific numbers of sessions of physical therapy can be found in S2 Appendix.

77% of patients had undergone at least one diagnostic procedure prior to visiting our clinic. A breakdown of number of patients who had each procedure is shown in Table 2. The three top diagnostics performed were Pelvic Ultrasound with 57% of patients having undergone one, Pelvic MRI with 40%, and exploratory surgery with 13%. In terms of highest utilization, 2 patients had reported having undergone 25 colonoscopies, 1 patient reported having undergone 30 esophagogastroduodenoscopies (EGD), and 1 patient reported having undergone 40 cystoscopies prior to visiting our practice. This analysis is shown in S3 Appendix.

**Table 2:** Average total cost burden of healthcare utilization to patients before presenting to our practice.

Procedures	CPT Code	Patients (N)	In-Network Cost per CPT code
**Treatments**			
Pelvic floor physical therapy *	90901	281	$ 368.00
**Average Cost per patient**			**$ 8,936.77**
**Diagnostics**			
Ultrasound	76830	263	$ 920
MRI	72197	184	$ 2,335
Exploratory Surgery	49320	57	$ 9,500
Urodynamics	51741	37	$ 291
Sigmoidoscopy	45330	25	$ 1,840
Semen Culture	89320	11	$ 202
Anal Ultrasound	76872	9	$ 720
Colonoscopy	45378	**	$ 5,348.00
Endoscopy	43235	**	$ 3,800.00
Cystoscopy	52000	**	$ 2,303.00
**Average Cost per patient**			**$ 5,264.22**
**Surgeries**			
Hysterectomy	58150	55	$ 25,048.00
Endometriosis excision	58662	50	$ 14,850.00
Endometriosis ablation	58563	29	$ 8,586.00
Oophorectomy	58661	25	$ 12,520.00
Ovarian cystectomy	58925	25	$ 16,730.00
Myomectomy	58545	14	$ 19,231.00
Pelvic floor repair	57425	7	$ 13,719.00
Bladder sling	57288	4	$ 11,118.00
Uterine artery embolization	37243	1	$ 17,892.00
Varicocelectomy	55550	2	$ 15,228.00
Testicular torsion surgery	54600	2	$ 8,725.00
Inguinal hernia repair	49650	22	$ 11,246.00
Hemorrhoid surgery	46260	17	$ 7,480.00
Colorectal surgery	44160	15	$ 6,685.00
Other hernia repair	49652	13	$ 14,655.00
Lumbar spine surgery	63030	11	$ 29,721.00
Hip FAI / Labral tear repair	29916	11	$ 18,559.00
**Average Cost per patient**			$ 15,750.37
**Total Cost Burden per patient**			**$ 29,951.36**

* Refer to S4 Appendix

** Refer to S3 Appendix

Out of 450 female patients in the pool, 137 reported having undergone at least one female specific surgery, representing 30% of the female participants. In total, 210 counts of female surgeries were reported. 58% of these patients had undergone an endometriosis related surgery, including endometriosis ablation or excision. The second highest undergone surgery was a hysterectomy, with 40% of this pool. Of the 157 male patients, 4 reported having undergone the male specific surgeries. Of the gender-neutral surgeries, 89 patients reported a past surgery. 25% of these patients had undergone an inguinal hernia repair, while the second highest surgery count was a hemorrhoid surgery with 20% of the patient pool (17 patients). Table 2 shows these results.

### Cost association of utilization of procedures

Costs are grouped in three categories: treatments, diagnostics, and surgeries.

#### Costs of treatment

Out of the 281 patients who had tried pelvic floor physical therapy and averaged 24 sessions each, the total cost burden was $8,937 per patient for in-network treatments. This was found by multiplying the average treatment sessions with the number of patients who tried pelvic floor physical therapy and the cost per CPT in-network as shown in Table 2.

#### Costs of diagnostics

The cost burden for the 466 patients who had undergone the diagnostics was $5,264.22. This is the average per person who have had at least one diagnostic. This was calculated by multiplying the number of patients associated with each diagnostic procedure and its cost per CPT code. For diagnostics which were undergone more than once, the cost per CPT code, number of patients, and frequency were multiplied. This can be found in S3 Appendix.

#### Costs of surgeries

The cost burden per patient for female specific surgeries was $16,800 for in-network services. For male specific surgeries, the cost burden was $11,977 for in-network services. The cost burden per patient for all other surgeries was $13,443 for in-network services. The overall cost burden per patient for all surgeries combined was $15,750 for in-network services.

#### Overall average costs

The overall cost burden per patient in this pool resulted as $29,951 for in-network services. This is divided into treatments, diagnostics, and surgeries as shown in Table 2.

**Fig 4:**
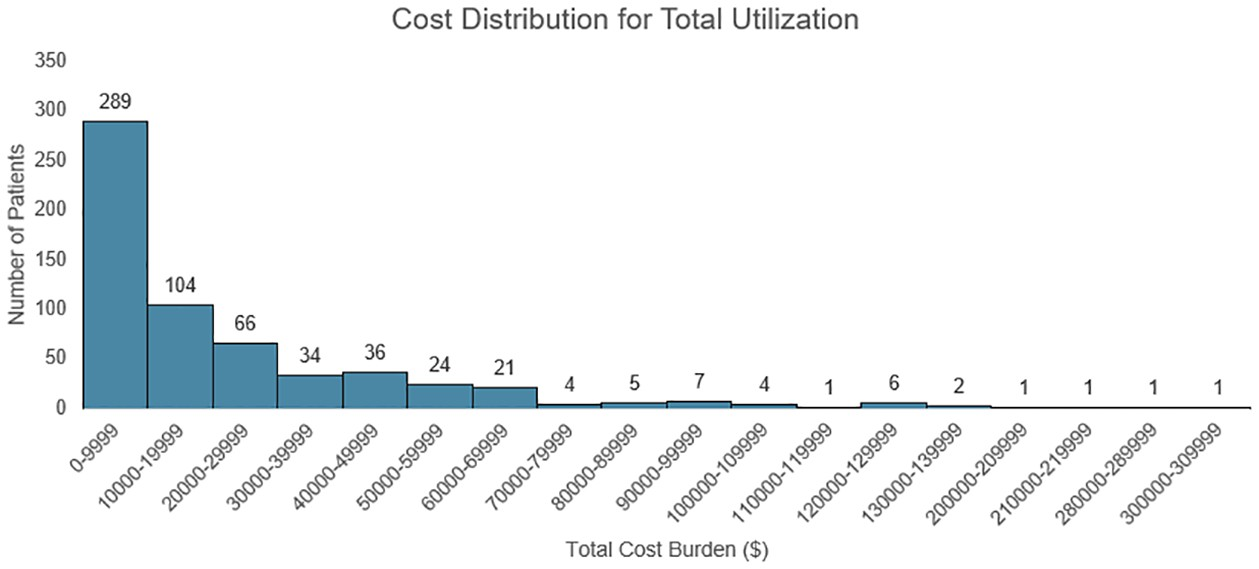
Cost distribution of inpatient services per patient before presenting to the practice.

A distribution of the costs incurred for in-network services by each patient encompassing treatment, diagnostics, and surgery costs is shown in Fig 4. A breakdown of this by category can be seen in S5 Appendix. The lowest total cost was incurred by 2 patients at $202 each for only undergoing the diagnostic of a Semen Culture. They reported experiencing pain for 5–10 years before presenting to us. The highest cost burden was $305,286. This patient reported having pain for more than 15 years and underwent 1 hysterectomy, 1 MRI, 1 exploratory surgery, 50 pelvic floor physical therapy sessions, 1 cystoscopy, 30 endoscopies, and 25 colonoscopies.

## Discussion

Our analysis of in-network costs to our patients totaled $29,951. This is not a yearly cost; it is the average cost CPP patients incur prior to consulting with us as their CPP persisted. The complex etiology of CPP and concomitant comorbidities and overlapping pain syndromes patients suffer from makes timely diagnosis and identification of effective treatments difficult. Patients often undergo multiple surgeries, multiple diagnostic invasive and non-invasive procedures, and present to multiple specialties thus incurring higher healthcare costs as found in our analysis. 14% of patients went to the Emergency Room seeking help for their pelvic pain symptoms. The majority of CPP patients are sent home from the Emergency Room with opioids and a referral to follow up with their OBGYN. This is not only a burden on healthcare utilization, but a significant disservice to CPP patients who are made worse by opioids [36, 37] and are looking for diagnostic expertise and a treatment plan. In addition to incurring healthcare utility costs, CPP patients also face a burden in their quality of life. In our patient pool, 76% were employed, of which 30.5% reported time lost from work and 62.1% reported reduced work productivity. A study by the Gallup Organization on women aged 18–50 who were suffering from CPP found that 14.7% of women endured pain for 3 months and reported significantly lower quality of life, pain from/ after intercourse, and loss of work time. Amongst 548 employed respondents, 15% reported time lost from paid work and 45% reported reduced work productivity [38]. Relating to presenteeism, a study of 193 women with endometriosis, CPP, and dysmenorrhea calculated 7.41 hours of work time lost per week [39]. Additionally, literature suggests patients with CPP tend to have a higher rate of anxiety and depression due to the onset of CPP which in turn affects intercourse and quality of life [40]. This is supported by our results as we found that among patients with at least one comorbidity, 71% had anxiety and depression. Among total patients, 66% had anxiety and depression. The presence of such comorbidities adds to the costs incurred by CPP patients. CPP patients with depression are prescribed opioids more often and at higher doses than non-depressed patients for example [38]. This further impacts their quality of life as repetitive use of opioids are not recommended for the treatment of CPP as it can cause opioid-induced constipation, narcotic bowel syndrome, and a rebound hyperalgesic effect on the central nervous system [37]. In our patient pool, 18% of patients had resorted to chronic opioid use. One of the reasons that worsen women’s quality of life with CPP, particularly with endometriosis, is the difficulties related to pregnancy and obstetric outcomes.

Negative effects on sexual function have been demonstrated in CPP. A clinical study was conducted on men with chronic pelvic pain to determine if sexual relations affect quality of life [44]. It concluded that sexual dysfunction is a key contributor to quality of life as men with sexual dysfunction had a significantly worse quality of life [41]. Women with CPP report greater levels of sexual displeasure and sexual complications than healthy controls [40].

Surgery may alleviate CPP in patients who have certain comorbidities such as endometriosis, adenomyosis, fibroids, pelvic organ prolapse, hernias or femoral-acetabular hip impingement/labral tear. Endometriosis is the most common pathologic cause of CPP in women of childbearing age. Though there are multiple types of Endometriosis, Deep Infiltrating Endometriosis (DIE), the most severe form of the disease affecting up to 5% of Endometriosis patients, and Ureteral Endometriosis (UE), which is found in up to 90% of patients alongside other sites affected by endometriosis, are the most costly to treat [42]. Patients see a high risk of recurrence of Endometriosis, which can multiply associated treatment costs. Recurrence rates for Deep Infiltrating Endometriosis (DIE) have been observed as high as 43.5%, and are highest when symptom recurrence noted is pain rather than surgical findings [43]. A retrospective study of 113,506 endometriosis patients concluded that two-thirds of them underwent an endometriosis related surgical procedure such as hysterectomy, laparoscopy, excision/ ablation, and oophorectomy [18]. Endometriosis related hysterectomies are responsible for 15%-18.9% of hysterectomies in the US [43, 44]. Out of the 55 hysterectomies in our patient pool, 44% were for endometriosis patients. However, as Endometriosis is found outside the uterus, a hysterectomy is not a definitive treatment for these patients [45]. Surgery accounts for 29% of health care costs for endometriosis patients while monitoring tests, hospitalization, and physician visits account for 19%, 18%, and 16% of costs respectively [16]. Furthermore, endometriosis related CPP congregates additional healthcare costs [46]. 71–80% of laparoscopies performed for women with CPP also had endometriosis. Laparoscopy’s direct surgical cost was estimated at $464 million per year in the US [47]. 98% of excisions, 100% of ablations, 44% of hysterectomies, and 56% of oophorectomies were undergone by women with endometriosis from our patient pool.

Adenomyosis is a benign uterine disorder with a prevalence ranging 8.8% to 61.5% depending on histopathologic diagnostic criteria. It also co-exists with conditions such as endometriosis (15–31%) and prolapse (20–31%) [48]. Adenomyosis is increasingly studied and revealing its associations with pelvic pain [49]. This disease detrimentally impacts quality of life through fertility, menstrual symptoms, and pregnancy outcomes therefore requires lifelong management [50]. Conservative surgical options, hysteroscopic resections/ablations, and uterine artery embolization are methods to treat adenomyosis surgically [51]. 18% of hysterectomies in our patient pool were undergone by women with adenomyosis. Hospital expenses for adenomyosis patients was found to have the highest cost compared to average gynecologic surgical expenses [52]. In our patient pool 5.6% of women reported adenomyosis. 72% of these women had concurrent endometriosis.

Uterine fibroids, the most common pelvic tumors range in prevalence from 4.5% to 68.6% due to genetic and environmental factors that affect certain populations [53, 54]. A survey of women diagnosed with fibroids showed that 71% of them use pharmacologic therapy and 30% underwent surgical procedures [55]. These surgical procedures contribute to the cost burden of $4–9.4 billion annually. The overall economic burden comprising of medications, surgery, and inpatient/outpatient visits is approximated at $5.9–24.4 billion yearly [56].

Pelvic organ prolapse can be treated through observation, pessaries, and surgery [57]. In 1997, direct costs for surgery were $1012 million. This includes vaginal hysterectomy ($494 million), cystocele and rectocele repair ($279 million), and abdominal hysterectomy ($135 million) [58]. Additionally, women with high risk of surgical failure require repeat surgery costing $2,298. Prevention of this via preoperative pelvic MRI increases costs by $90 million while avoiding 39,150 surgical failures [59]. In our patient pool, 7% of women had pelvic floor prolapse and 22% of them had undergone a pelvic floor repair.

Hernia repair surgeries are estimated at 600,000 surgeries annually in the United States [60]. Potential deleterious complications of hernia surgeries include bladder injury, recurrence, wound infection, and chronic neuropathic pain [61] which further contribute to medical costs. A longitudinal study reporting total costs on patients 9 years post-hernia repairs found $37,388 as the average total inpatient/ outpatient cost for patients who did not develop chronic pain. This cost increased to $51,334 for patients who developed chronic pain [60]. In our patient pool, 11% of patients had a hernia and 35 patients underwent hernia repair before presenting to us.

Hip arthroscopy is a frequent surgical procedure used to treat femoral-acetabular hip impingement and labral tears which is seen in the CPP population as groin pain is a common chief complaint [62]. It costs $2653 more than structured rehabilitation alone and reduces in cost-effectiveness with increasing age [63]. In our patient pool, 7% of patients underwent hip femoroacetabular impingement surgery.

This analysis used in-network costs obtained from the FAIR Health Consumer online database. The cost estimates therefore do not include government programs such as Medicare, Medicaid, or military plans. Since a geographic location is required to retrieve cost estimates per CPT code, our analysis was based only on one location: 10001 (New York, New York). Since our patient population is from large metropolitan areas, with New York, New York being the majority, using only this location gives an accurate account of what costs patients incurred in large metropolitan areas before presenting to us. To generalize the results from our analysis, care must be taken to which geographic location providers or patients reside in.

Another limitation of this cost burden analysis is the lack of data on costs of medications that patients have tried prior to presenting to us and costs associated with work hours lost. We also only chose pelvic floor physical therapy as the treatment we analyzed rather than analyzing costs of all other treatments tried. The average cost per patient would be much higher if we included this data. Future publications with this data are warranted to get an even more accurate representation of the cost burden incurred by CPP patients.

## Conclusion

In conclusion, Chronic Pelvic Pain was found to have a large cost burden of $29,951 for in-network services which includes treatments, diagnostics, and surgeries. These in-network services are often redundant and unnecessary. This leads to an increase in healthcare costs for CPP patients while simultaneously negatively affecting their quality of life and functional capacity. This publication highlights the societal and individual cost burden of CPP in order to encourage physicians to understand CPP and its multifaceted complex nature so that patients can be treated with better outcomes and minimize the costs associated with systematic healthcare utilization from delayed diagnosis and treatment. A streamlined interdisciplinary multimodal approach to a more rapid diagnosis and treatment of CPP patients with a clearly delineated treatment algorithm will improve the quality of life of CPP patients and decrease the current dis-utilization of the healthcare system.

## Supporting information

S1 Appendix System based causes and comorbidities of Chronic Pelvic Pain. (DOCX)

S2 Appendix Utilization of pelvic floor physical therapy. (DOCX)

S3 Appendix Analysis of diagnostic procedures with frequency of more than 1. (DOCX)

S4 Appendix Cost association of utilization of pelvic floor physical therapy. (DOCX)

S5 Appendix Cost distributions by treatments, diagnostics, and surgeries. (DOCX)

S1 Dataset (XLSX)
