# Supplementary material for: The burden of Chronic Pelvic Pain (CPP): Costs and quality of life of women and men with CPP treated in outpatient referral centers
Source: PLoS One. 2023 Feb 9;18(2):e0269828. doi: 10.1371/journal.pone.0269828 (PMC9910684; doi:10.1371/journal.pone.0269828)
Supplement: S1 Appendix — (DOCX) [file pone.0269828.s001.docx]

**S1 Appendix A.** System based causes and comorbidities of Chronic Pelvic Pain.

| **Systems** | **Conditions** | |
| --- | --- | --- |
| **Reproductive** | **Female** | **Male** |
|  | Adenomyosis  Adhesions  Cancer  Dermatosis  Endometriosis  Fibroids  Ovarian Remnant Syndrome  Ovarian Retention  Pelvic Inflammatory Disease  Polycystic Ovarian Syndrome  Vaginitis  Vulvodynia  Vulvovaginitis | Chronic Epididymitis  Chronic Orchalgia  Cancer |
| **Urological** | Bladder Pain Syndrome/ Interstitial Cystitis  Bladder Dysfunction  Cancer  Chronic Non-Bacterial Prostatitis (UCPPS)  Prostatodynia | |
| **Gastrointestinal** | Cancer  Hernia (Inguinal, Umbilical, Obturator)  Irritable Bowel Syndrome (Crohn's and Ulcerative colitis)  Inflammatory Bowel Disease  Recurrent Small Bowel Obstruction  Small Intestinal Bacterial Overgrowth (SIBO) | |
| **Musculoskeletal** | Abdominal Myofascial Pain Syndrome  Cancer  Diastasis Recti  External Rotators Hip Tension Myalgia  Femoroacetabular Impingement (FAI) /Hip Labral Tear  Lumbosacral Degenerative Disc Disease/Facet Arthrosis  Pelvic Floor Muscle Tension Myalgia  Pubic Symphysis Dysfunction  Sacroiliac Joint Dysfunction  Sports Hernia | |
| **Vascular** | Cancer  Pelvic Congestion Syndrome | |
| **Neurologic** | Abdominopelvic neuralgias (ilioinguinal, iliohypogastric, genitofemoral, obturator, lateral femoral cutaneous, pudendal, posterior femoral cutaneous, inferior cluneal)  Cancer  Lumbosacral Plexopathy  Lumbosacral Radiculopathy  Neuralgia/Cutaneous Nerve Entrapment of Ilioinguinal/Iliohypogastric/Genital Branch of Genitofemoral Nerve  Pudendal Neuralgia  Shingles (Postherpetic Neuralgias (HSV1 and HSV2 infections)) | |
| **Rheumatologic** | Auto-Immune Polyradiculopathy  Cancer  Fibromyalgia  Rheumatoid Arthritis | |
| **Psychosocial** | Abuse  Anxiety  Depression  PTSD | |
