# Supplementary material for: The burden of Chronic Pelvic Pain (CPP): Costs and quality of life of women and men with CPP treated in outpatient referral centers
Source: PLoS One. 2023 Feb 9;18(2):e0269828. doi: 10.1371/journal.pone.0269828 (PMC9910684; doi:10.1371/journal.pone.0269828)
Supplement: S2 Appendix — (DOCX) [file pone.0269828.s002.docx]

**S2 Appendix B.** Utilization of Pelvic floor physical therapy

| **Number of Pelvic Floor Physical Therapy Sessions tried** | **Number of Patients** |
| --- | --- |
| 0-9 | 444 |
| 10-19 | 66 |
| 20-29 | 35 |
| 30-39 | 18 |
| 40-49 | 9 |
| 50-59 | 11 |
| 60-69 | 6 |
| 70-79 | 4 |
| 100-109 | 7 |
| 120-129 | 1 |
| 150-159 | 1 |
| 200-209 | 2 |
| 250-259 | 1 |
| 410-419 | 1 |
| 490-500 | 1 |
