# Supplementary material for: The burden of Chronic Pelvic Pain (CPP): Costs and quality of life of women and men with CPP treated in outpatient referral centers
Source: PLoS One. 2023 Feb 9;18(2):e0269828. doi: 10.1371/journal.pone.0269828 (PMC9910684; doi:10.1371/journal.pone.0269828)
Supplement: S3 Appendix — (DOCX) [file pone.0269828.s003.docx]

**S3 Appendix C.** Analysis of Diagnostic procedures with frequency of more than 1.

| **Diagnostics** | **CPT Code** | **Frequency** | **Patients (N)** | **In-Network Cost per CPT** |
| --- | --- | --- | --- | --- |
| Colonoscopy | 45378 | 1 | 146 | $ 5,348.00 |
|  |  | 2 | 41 | " |
|  |  | 3 | 19 | " |
|  |  | 4 | 12 | " |
|  |  | 5 | 12 | " |
|  |  | 6 | 1 | " |
|  |  | 7 | 4 | " |
|  |  | 8 | 1 | " |
|  |  | 10 | 2 | " |
|  |  | 25 | 2 | " |
| **Average Cost per patient** | |  |  | $ 11,253.08 |
| Endoscopy | 43235 | 1 | 136 | $ 3,800.00 |
|  |  | 2 | 43 | " |
|  |  | 3 | 7 | " |
|  |  | 4 | 6 | " |
|  |  | 5 | 5 | " |
|  |  | 6 | 2 | " |
|  |  | 7 | 1 | " |
|  |  | 10 | 2 | " |
|  |  | 20 | 1 | " |
|  |  | 30 | 1 | " |
| **Average Cost per patient** | |  |  | $ 7,097.06 |
| Cystoscopy | 52000 | 1 | 75 | $ 2,303.00 |
|  |  | 2 | 32 | " |
|  |  | 3 | 10 | " |
|  |  | 4 | 2 | " |
|  |  | 5 | 3 | " |
|  |  | 6 | 1 | " |
|  |  | 7 | 1 | " |
|  |  | 8 | 2 | " |
|  |  | 10 | 1 | " |
|  |  | 12 | 2 | " |
|  |  | 40 | 1 | " |
| **Average Cost per patient** | |  |  | $ 5,226.04 |
