# Supplementary material for: The burden of Chronic Pelvic Pain (CPP): Costs and quality of life of women and men with CPP treated in outpatient referral centers
Source: PLoS One. 2023 Feb 9;18(2):e0269828. doi: 10.1371/journal.pone.0269828 (PMC9910684; doi:10.1371/journal.pone.0269828)
Supplement: S4 Appendix — (DOCX) [file pone.0269828.s004.docx]

**S4 Appendix D** Cost association of utilization of pelvic floor physical therapy

| **Treatment** | **CPT Code** | **Average number of sessions** | **Patients (N)** | **In-Network Cost per CPT** |
| --- | --- | --- | --- | --- |
| Pelvic floor physical therapy | 90901 | 24.28 | 281 | $368.00 |
| **Average Cost per patient** |  |  |  | $8,936.77 |
