# Supplementary material for: The burden of Chronic Pelvic Pain (CPP): Costs and quality of life of women and men with CPP treated in outpatient referral centers
Source: PLoS One. 2023 Feb 9;18(2):e0269828. doi: 10.1371/journal.pone.0269828 (PMC9910684; doi:10.1371/journal.pone.0269828)
Supplement: S5 Appendix — (DOCX) [file pone.0269828.s005.docx]

**S5 Appendix E.** Cost distributions by treatments, diagnostics, and surgeries
